# DAP12 and CD11b contribute to the microglial-induced death of dopaminergic neurons *in vitro* but not *in vivo* in the MPTP mouse model of Parkinson’s disease

**DOI:** 10.1186/1742-2094-10-82

**Published:** 2013-07-11

**Authors:** Kiyoka Kinugawa, Yann Monnet, Catherine Béchade, Daniel Alvarez-Fischer, Etienne C Hirsch, Alain Bessis, Stéphane Hunot

**Affiliations:** 1 CNRS, UMR 7225, Experimental Therapeutics of Neurodegeneration, Paris F-75013, France; 2 UPMC Univ Paris 06, UMR_S975, Paris F-75013, France; 3 INSERM, UMR_S975, CRICM, Paris F-75013, France; 4 Assistance Publique-Hôpitaux de Paris (AP-HP), Functional Explorations Unit of the Elderly, Charles Foix Hospital, Ivry-sur-Seine F-94200, France; 5 Institut de Biologie de l’Ecole Normale Supérieure, Paris F-75005, France; 6 INSERM U1024, Paris F-75005, France; 7 CNRS, UMR 8197, Paris F-75005, France; 8 Department of Neurology, Philipps-University Marburg, Marburg 35043, Germany; 9 Institute of Neurogenetics, University of Lübeck, Lübeck, Germany; 10 Department of Psychiatry, University of Lübeck, Lübeck, Germany; 11 Centre de Recherche ICM, INSERM/UPMC UMR S975, CNRS UMR 7225, Hôpital de la Pitié-Salpêtrière, 47 Bd de l’Hôpital, Bat. ICM, 5e étage, Paris F-75005, France

**Keywords:** DAP12, CD11b, Microglia, Dopaminergic neuron, Parkinson’s disease, MPTP

## Abstract

**Background:**

Parkinson’s disease (PD) is a neurodegenerative disorder characterized by a loss of dopaminergic neurons (DN) in the substantia nigra (SN). Several lines of evidence suggest that apoptotic cell death of DN is driven in part by non-cell autonomous mechanisms orchestrated by microglial cell-mediated inflammatory processes. Although the mechanisms and molecular network underlying this deleterious cross-talk between DN and microglial cells remain largely unknown, previous work indicates that, upon DN injury, activation of the β2 integrin subunit CD11b is required for microglia-mediated DN cell death. Interestingly, during brain development, the CD11b integrin is also involved in microglial induction of neuronal apoptosis and has been shown to act in concert with the DAP12 immunoreceptor. Whether such a developmental CD11b/DAP12 pathway could be reactivated in a pathological context such as PD and play a role in microglia-induced DN cell death is a tantalizing hypothesis that we wished to test in this study.

**Methods:**

To test the possibility that DAP12 could be involved in microglia-associated DN injury, we used both *in vitro* and *in vivo* toxin-based experimental models of PD recapitulating microglial-mediated non-cell autonomous mechanisms of DN cell death. *In vitro*, enriched mesencephalic neuronal/microglial co-cultures were exposed to the dopaminergic neurotoxin 1-methyl-4-phenylpyridinium (MPP+) whereas *in vivo*, mice were administrated with 1-methyl-4-phenyl-1,2,3,6-tetrahydropyridine (MPTP) according to acute or subchronic mode. Mice deficient for DAP12 or CD11b were used to determine the pathological function of the CD11b/DAP12 pathway in our disease models.

**Results:**

Our results show that DAP12 and CD11b partially contribute to microglia-induced DN cell death *in vitro*. Yet, *in vivo*, mice deficient for either of these factors develop similar neuropathological alterations as their wild-type counterparts in two different MPTP mouse models of PD.

**Conclusion:**

Overall, our data suggest that DAP12 and CD11b contribute to microglial-induced DN cell death *in vitro* but not *in vivo* in the MPTP mouse model of PD. Therefore, the CD11b/DAP12 pathway may not be considered as a promising therapeutic target for PD.

## Background

Parkinson’s disease (PD) is a neurodegenerative disorder characterized by a loss of dopaminergic neurons (DN) in the substantia nigra (SN). Although the mechanism by which these neurons degenerate is still uncertain, several lines of evidence suggest that apoptotic cell death of DN is driven in part by non-cell autonomous mechanisms implicating microglial cells and inflammatory processes [1]. Current hypothesis suggests that DN injury initiated by as-yet to be identified etiological factors triggers microglial cell activation, which in turn set off harmful inflammatory mechanisms involved in a vicious cycle of neuronal cell death. Among the different scenarios underlying microglial neurotoxicity during PD-like nigrostriatal pathway injury, the production of toxic levels of reactive oxygen (ROS) and nitrogen species by activated microglial cells is believed to be a major threat for DN survival [1]. This view is supported by experimental findings showing that genetic ablation of some of the key enzymatic systems involved in macrophage-associated respiratory burst (inducible nitric oxide synthase and nicotinamide adenine dinucleotide phosphate-oxidase (NADPH)-oxidase) provides neuroprotection in animal models of PD [2,3].

The molecular signals from the dying/suffering DN neurons that trigger microglial cell activation and the oxidative burst are still largely unknown. Yet, recent evidence indicate that microglial CD11b, the alpha M subunit of the MAC1 β2 integrin, is likely to be involved in NADPH-oxidase activation and superoxide production [4,5]. This process is reminiscent to the mechanisms used by peripheral macrophages and neutrophils to trigger the death of invading pathogens through CD11a- or CD11b-mediated ROS production [6,7]. In this context, β2 integrin signaling has been shown to proceed by an immunoreceptor-like mechanism that involves adaptors containing immunoreceptor tyrosine-based activation motifs (ITAMs) including DAP12 and FcRγ [8]. With respect to the CNS, a CD11b/DAP12 pathway has been previously implicated in the induction of developmental neuronal apoptosis by microglial cells in the hippocampus [9]. Although still debated, DAP12 expression is usually undetected or poorly detected in the adult brain and has been shown to be restricted to developing microglia [10,11]. Whether such developmental pathway of neuronal cell death could be reactivated in adulthood under specific neuropathological circumstances such as PD is an open question.

To test this hypothesis, we used both *in vitro* and *in vivo* models of PD recapitulating microglia-mediated non-cell autonomous mechanisms of DN neurodegeneration. We found that whereas DAP12 and CD11b partially contribute to microglia-induced DN cell death *in vitro*, mice deficient for either of these factors develop similar neuropathological alterations to their wild-type (WT) counterparts in two different 1-methyl-4-phenyl-1,2,3,6-tetrahydropyridine (MPTP) mouse models of PD.

## Methods

### Animals

C57BL/6J mice (Janvier Breeding Center, Le Genest St Isle, France), DAP12-mutated mice (DAP12^KI^) on C57BL/6 genetic background [12] and CD11b-deficient mice (B6.129S4-Itgam^tm1Myd^/J on C57BL/6 background obtained from the Jackson Laboratory) were used. For *in vitro* experiments, primary mesencephalic neurons were prepared from gestational age 13 days C57BL/6J mice embryos whereas primary microglial cells were obtained from newborn C57BL/6J, DAP12^KI^ or CD11b^−/−^ mice. For *in vivo* experiments, ten- to twelve week-old male C57BL/6J, DAP12^KI^ and CD11b^−/−^ mice, weighing 25 to 30 g were used. Mice were kept in a temperature-controlled room (23°C ± 1°C) under a 12-hour light/dark cycle and had *ad libitum* access to food and water. All animals were further genotyped after their sacrifice. Animal handling was carried out according to ethical regulations and guidelines (Guide for the care and use of laboratory animals, NIH publication no. 85–23, revised 1985) and the European Communities Council Directive 86/609/EEC. Experiments using vertebrates were approved by the Services Vétérinaires de Paris.

### Pharmacological agents

Lipopolysaccharide (LPS),1-methyl-4-phenylpyridinium (MPP^+^) and 1-methyl-4-phenyl-1,2,3,6-tetrahydropyridine hydrochloride (MPTP-HCl) were purchased from Sigma-Aldrich (St Quentin-Fallavier, France). Dihydrorhodamine-123 (DHR-123), the cell permeant probes used for the detection of ROS was purchased from Molecular Probes (Invitrogen, CergyPontoise, France).

### Midbrain cell cultures

Cultures were prepared from the ventral mesencephalon of gestational age 13 days C57BL/6J mice embryos. Dissociated cells in suspension obtained by mechanical trituration of midbrain tissue pieces were seeded at a density of 1.2 to 1.5 × 10^5^ cells/cm^2^ onto tissue culture supports pre-coated with 1 mg/mL polyethylenimine (Sigma-Aldrich, St Quentin-Fallavier, France). The cultures were then maintained in N5 medium supplemented with 5 mM glucose, 5% horse serum, and 0.5% fetal calf serum, except for the first 3 days *in vitro* (DIV) during which the concentration of fetal calf serum was set at 2.5% to favor initial maturation of the cultures [13]. Note that tyrosine hydroxylase-positive (TH^+^) neurons represent approximately 1 to 2% of the total number of neuronal cells present in these cultures.

### Highly enriched microglial cell cultures

Microglial cells were derived from the cerebral cortex of newborn C57BL/6J, DAP12^KI^ or CD11b^−/−^ mice, according to procedures described previously [14]. Pure ameboid microglial cells (>99%) were isolated from 2-week-old primary glial cultures grown in DMEM supplemented with 10% fetal calf serum. The cells were washed three times in DMEM and plated in CDM (1 to 1.5 × 10^6^ cells per 35 mm dish).

### Mixed neuron/microglia cultures

Freshly isolated microglial cells (1 to 2.10^5^ cells/well) were added to 4-DIV primary neuronal cultures grown in 24-well plates. Control neuronal cultures were supplemented with an equal volume of cell-free medium. LPS (serotype 026:B6, *Escherichia coli*, at 100 ng/mL) or MPP^+^, at 0.1 μM, were applied directly to the mixed cultures at 5-DIV, that is, one day after the seeding of microglial cells on primary neurons.

### Immunofluorescent detection protocols

The cultures were fixed for 12 minutes using 4% formaldehyde in Dulbecco’s (PBS), and then washed twice with PBS before an incubation step at 4°C for 24 to 72 h with the following antibodies. A monoclonal anti-TH antibody diluted 1/5000 (Diasorin, Stillwater, MN, USA) was used to assess the survival of DN. Microglial cells were characterized using a mouse anti-CD11b antibody (1/50; clone MRC OX-42; Serotec, Oxford, England). All antibodies were diluted in PBS containing 0.2% Triton X-100 except the mouse anti-CD11b antibody, which was diluted in PBS only. Detection of the primary antibodies was performed with a cyanin-3 conjugate of an anti-mouse IgG antibody (1/500, Sigma Aldrich) or with an Alexa Fluor 488 conjugate of an anti-rabbit antibody (1/500; Invitrogen, Carlsbad, CA, USA). Cell counting was performed at 200× magnification using a 20× objective matched with a 10× ocular. The number of TH^+^ neurons in each culture well was estimated after counting 20 visual fields distributed along the X and Y axes. Note that counts of neuronal cells were performed at 13-DIV, that is, at a stage when the death process affecting DA neurons is almost fully complete.

### Quantification of superoxide ions

Superoxyde ion (O_2_^.-^) levels were measured using dihydroethidium (Invitrogen) as fluorescent probes at 6-DIV, that is, when oxidative stress is at its peak. Mixed microglia-neuron cultures were exposed for 30 minutes to 1 μM dihydroethidium, washed three times, and then maintained in serum-free supplemented medium. The fluorescent signal, visualized by epifluorescence microscopy (excitation at 520 nm, emission at 610 nm), was quantified using the Simple-PCI software from C-Imaging Systems (Cranberry Township, PA, USA) and a Nikon TE-300 inverted microscope equipped with an ORCA-ER digital camera (Hamamatsu Photonics, Massy, France). Fluorescent images of randomly chosen fields (six to eight in each culture condition) were acquired with a 20× fluorescent objective. The average pixel intensity over the surface of each cell body was determined under the different test conditions. Background fluorescence was subtracted from raw data, and the results were expressed as a percentage of the mean fluorescence intensity per cell in control cultures. A minimum of 60 cells was analyzed under each test condition.

### MPTP injection and tissue preparation

Groups of mice received MPTP under a subchronic or acute protocol. For subchronic MPTP intoxication, mice were given an intra-peritoneal (i.p.) injection of 30 mg/kg (free base) MPTP-HCl per day for 5 consecutive days and were then euthanized at 1, 2, 4, 7 or 21 days after the last MPTP injection. For acute MPTP intoxication, mice were i.p.-injected with 4 doses of 20 mg/kg (free base) MPTP-HCl at 2-h intervals and were then euthanized 2 or 7 days after the last MPTP injection. Control mice received an equivalent volume of 0.9% NaCl solution. For immunohistochemistry, mice were injected with a lethal dose of pentobarbital (100 mg/kg) and then transcardially perfused with 50 mL of heparin solution (5 U/mL) followed by 100 mL of ice-cold 4% paraformaldehyde (PFA) solution. After extraction from the skull, brains were further post-fixed overnight in fresh 4% PFA/PB solution, and cryoprotected with 30% sucrose in PB. Coronal free-floating striatal and mesencephalic sections (30-μm thick) were prepared using a freezing microtome (Leica) and collected in 10 regularly spaced series. For quantitative PCR, brains were rapidly removed from the skull and striata, ventral mesencephalon and cortex were dissected on humidified filters at 4°C. Tissues were then frozen in liquid nitrogen and kept at −80°C until use.

### Real-time polymerase chain reaction (PCR)

Real-time quantitative (q)PCR was performed as described [15]. The primer sequences were as follows: mouse *Dat* forward 5-CGC TGG AGG CAG TCG AA-3, and reverse 5-CGG AGC ATT TGC TTT TAC TCA TG-3; mouse *Cd11b* forward 5-GAT GCT TAC CTG GGT TAT GCT TCT-3, and reverse 5-CCG AGG TGC TCC TAA AAC CA-3; mouse *Dap12* forward 5-TGG TGT TGA CTC TGC TGA TTG C-3, and reverse 5-CCT TCC GCT GTC CCT TGA C-3. Primer sequences of housekeeping gene were as follow: mouse *Hprt* forward 5-CTT CCT CCT CAG ACC GCT TTT-3, and reverse 5-AAC CTG GTT CAT CAT CGC TAA TC-3; mouse *Gapdh* forward 5-TGT GTC CGT CGT GGA TCT GA-3, and reverse 5-CCT GCT TCA CCA CCT TCT TGA-3.

### Measurement of striatal MPP^+^ levels

Mice were euthanized 90 minutes after one i.p. injection of 30 mg/kg MPTP-HCl, and their striata were recovered and treated with 500 μL 0.1 N HClO4 before being processed for HPLC using UV detection (295-nm wavelength).

### Measurement of striatal dopamine, DOPAC and HVA levels

Seven days after the last MPTP injection, mice were euthanized and their striata were dissected out and treated with 0.1 N perchloric acid containing 0.05% disodium ethylenediaminetetraacetic acid (EDTA) and 0.05% sodium metabisulfite. Striatal tissue content in dopamine, 3,4-dihydroxyphenylacetic acid (DOPAC) and homovanillic acid (HVA) was assessed by high performance liquid chromatography (HPLC) using electrochemical detection with a potential set at +0.65 V.

### Immunohistochemistry

Immunohistochemical staining on mouse brain sections was performed as previously described [16]. The following primary antibodies were used: anti-TH (1:1000; Pel-Freez Biochemicals), anti-Iba1 (1:500; Wako Chemicals). Staining was revealed by the ABC method (Vector Laboratories) with 3,3-diaminobenzidine (DAB) as the peroxidase substrate. Mouse sections were counterstained with thionin solution (Nissl stain).

For double-staining experiments, brain sections were simultaneously incubated with two primary antibodies: anti-CD11b (rat - 1:250; Serotec, Oxford, England), anti-DAP12 (rabbit- 1:100; Chemicon International, Merk Millipore, Molsheim, France). Sections were then incubated in specific CY3- or Alexa488-conjugated secondary antibodies (Jackson ImmunoResearch Europe, Suffok, England) at 1:1000 dilution for 120 minutes at room temperature.

### Image and data analysis

DAB-immunostained sections were analyzed by bright-field microscopy, using a Leitz microscope equipped with image analysis software (Mercator, ExploraNova, La Rochelle, France). TH^+^ and Nissl^+^ cell bodies were quantified stereologically on regularly spaced sections covering the whole substantia nigra *pars compacta* (SNpc) using the VisioScan stereology tool. The investigator performing the quantification was blinded to the treatment and genotype groups during the analysis. Fluorescent sections were analyzed on a Zeiss Axioplan 2 using ExploraNova FluoUp 1.0 software. Striatal TH optic densitometry was measured by image analysis software (Mercator, ExploraNova).

### Statistics

All values are expressed as the mean ± standard error of the mean (SEM). Differences in means between two groups were analyzed using the two-tailed Student’s *t*-test, or when data were not normally distributed, with the nonparametric Mann–Whitney *U*-test. Differences in means among multiple datasets were analyzed using one- or two-way analysis of variance (ANOVA) with time, treatment, or genotype as the independent factors. When ANOVA showed significant differences, pairwise comparisons between means were tested by the Tukey post hoc analysis. When data were not normally distributed, ANOVA on ranks was used (Kruskal-Wallis test followed by pairwise comparison using the Dunn test). In all analyses, *P*-values of less than 0.05 were considered significant (SigmaStat Statistical Software, Systat Software, Inc., San Jose, CA, USA).

## Results

### Microglial DAP12 contributes to the LPS-induced dopaminergic neurotoxicity *in vitro*

**Figure 1:**
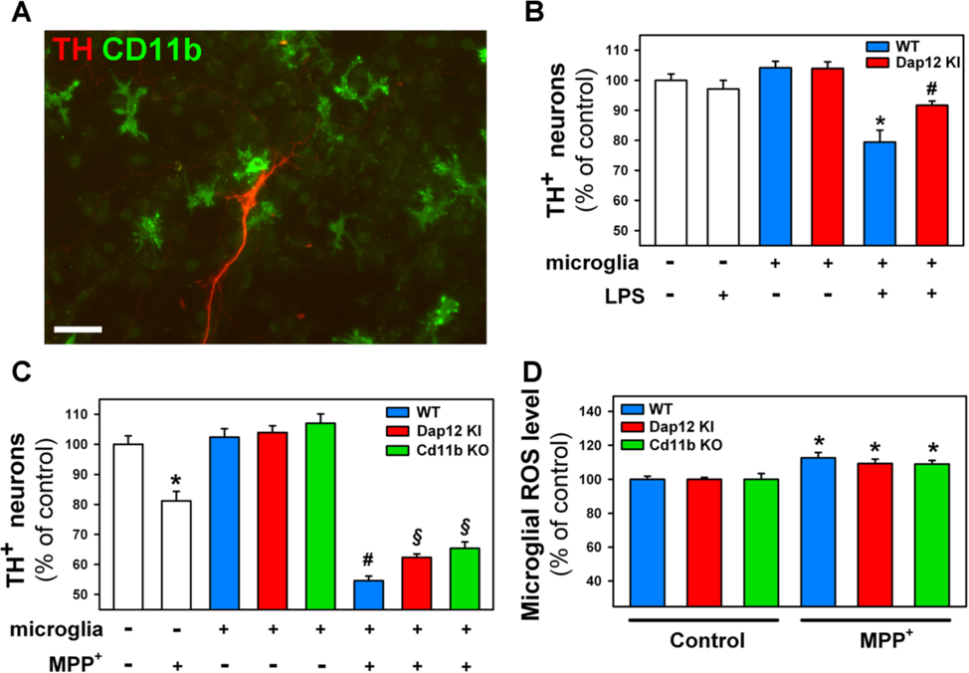
**Microglial Dap12 and CD11b contribute to lipopolysaccharide (LPS)- and 1-methyl-4-phenylpyridinium (MPP**^**+**^**)-induced dopaminergic neurotoxicity *in vitro*. (A)** Representative image showing tyrosine hydroxylase (TH)-expressing dopaminergic neurons (red) co-cultured with CD11b-positive microglial cells (green). Scale bar: 50 μm. **(B)** Quantification of TH^+^ neurons in LPS-treated neuronal cultures with or without microglial cells. In LPS-treated neuronal cultures, addition of wild-type (WT) microglial cells (blue bar) results in significant neurotoxicity as compared to untreated cultures with or without WT microglia. LPS-induced neurotoxicity is attenuated in the presence of DAP12-deficient (Dap12 KI) microglia (red bar) compared to WT microglia. Data are expressed as mean ± standard error of the mean (SEM). ^*^P <0.01 compared to untreated mixed cultures; ^#^*P* <0.05 compared to LPS-treated mixed cultures with WT microglia. **(C)** Quantification of TH^+^ neurons in MPP^+^-treated neuronal cultures in the presence or absence of microglial cells from WT (blue bar), Dap12 KI (red bar) or Cd11b KO (green bar) mice. In pure neuronal cultures, MPP^+^ induces a 20% TH^+^ cell loss. ^*^P <0.001 compared to untreated neuronal cultures. Addition of WT microglial cells in MPP^+^-treated cultures further increases dopaminergic cell death (45% neuronal loss). In neuron/microglia co-cultures, MPP^+^-induced neurotoxicity is attenuated in the presence of either DAP12- or CD11b-deficient microglia compared to WT microglia. ^#^*P* <0.01 compared to untreated mixed neuron/microglia and pure neuronal cultures; ^§^*P* <0.05 compared to MPP^+^-treated mixed cultures with WT microglia. **(D)** Quantification of microglia-derived reactive oxygen species (ROS) level in mixed neuron/microglia cultures under basal condition or 24 h after MPP^+^ exposure. Treatment of mixed neuron/microglia cultures with MPP^+^ results in a rise in microglial-derived ROS whose levels were of similar magnitude between microglial genotypes. *P <0.01 compared to their respective untreated mixed cultures (Dunn test).

The involvement of the microglial DAP12 and CD11b proteins in the pathological induction of DN death was first investigated *in vitro* using mixed cultures of mesencephalic neurons and microglial cells (Figure 1A). In these cultures, the survival of DA neurons was assessed by measuring the number of neurons expressing TH, the rate-limiting enzyme of the dopamine synthesis pathway.

The number of TH-immunoreactive neurons was not significantly different when mesencephalic neurons were cultured in the absence or in the presence of non-stimulated microglia (Figure 1B). This showed that in this co-culture system, non-stimulated microglia do not induce DN death. By contrast, when mesencephalic neurons were cultured with WT microglia stimulated by LPS (100 ng/mL), the number of TH-expressing neurons decreased by 20.6% ± 3.9% (*P* = 0.0096, Dunn test; Figure 1B). Addition of LPS alone to DN cultures was not neurotoxic (98.5% ± 3.7%, *P* = 0.732, Dunn test; Figure 1B), showing that the toxic effect of LPS is not cell-autonomous. These experiments show that, as reported previously [17], LPS-activated microglia are toxic for DN.

It has been shown that CD11b is involved in LPS-induced neurotoxicity of microglia [4]. In addition, CD11b acts in synergy with DAP12 to induce the developmental neuronal death in the hippocampus [9]. Based on these data, we investigated the role of DAP12 in LPS-induced dopaminergic neurotoxicity of microglia. Figure 1B shows that activation of DAP12-deficient microglia with LPS caused significantly less DN death as compared to WT microglia (8.3% ± 1.5%; *P* = 0.015, Dunn test). These results show that like CD11b, DAP12 contributes also to the LPS-induced neurotoxicity of microglia.

### Microglial DAP12 and CD11b contribute to the MPP^+^-induced dopaminergic neurotoxicity *in vitro*

We next investigated the role of microglial DAP12 and CD11b in a more relevant model of PD-associated dopaminergic cell death. We thus cultured mesencephalic neurons alone or with WT, DAP12- or CD11b-deficient microglia and monitored the selective death of DN induced by the neurotoxin MPP^+^[18]. As expected, MPP^+^ treatment of mesencephalic neuronal cultures induces, on average, a 20% DN cell loss (Figure 1C). When mesencephalic neurons were co-cultured with WT microglia, MPP^+^ induced stronger loss of TH-expressing cells than when DN were cultured alone (45.3% ± 2.1%, *P* = 0.004, Dunn test) (Figure 1C). Noteworthy, when mesencephalic neurons were co-cultured with DAP12- or CD11b-deficient microglia, application of MPP^+^ induced significantly less death compared to WT microglial cells (DAP12^KI^: 37.8% ± 0.9%, *P* = 0.035; CD11b^−/−^: 34.8 ± 1.5%, *P* = 0.012, Dunn test). These results show that microglial DAP12 and CD11b participate in non-cell autonomous mechanisms of DN cell death induced by MPP^+^.

In some systems, the mechanism of neuronal death induced by activated microglial cells involves the CD11b-dependent production of ROS [4,9]. This raises the possibility that in mesencephalic neuronal cultures, the death of DN induced by MPP^+^-stimulated microglia is mediated, at least in part, by the DAP12- and CD11b-dependent production of ROS. To test this hypothesis, we first established an assay to measure microglial cell-derived ROS production. We took advantage of the property of O_2_^.-^ to specifically oxidize dihydroethidium (DHE) to ethidium, which then binds to the nucleic acids in the cells in which it has been produced [19]. We thus measured the production of ROS by WT, DAP12-deficient and CD11b-deficient microglia co-cultured with mesencephalic neurons challenged or not with MPP^+^. As expected, exposure of neuron/microglia co-cultures with MPP^+^ induced a significant increase in the intensity of ethidium production by microglial cells (17.2% ± 1.3%, *P* = 0.006, Dunn test) (Figure 1D). Yet, the same increase was detected when DAP12- or CD11b-deficient microglia were co-cultured with WT neurons (DAP12 KI: 15.1% ± 0.9%, *P* = 0.752; CD11b KO: 15.0% ± 0.7%, *P* = 0.261 compared to WT microglia; Dunn test). This result shows that in contradiction with a previous *in vitro* study [5], the production of ROS following stimulation of microglia by MPP^+^-induced DN injury is not dependent on DAP12 or CD11b.

### Increased DAP12 and CD11b expression in the substantia nigra parallels DA neurons degeneration upon 1-methyl-4-phenyl-1,2,3,6-tetrahydropyridine (MPTP) intoxication

Our *in vitro* data show that DAP12 and CD11b contribute to the toxicity of microglia stimulated by MPP^+^-induced dopaminergic injury. These results suggest that microglial DAP12 and CD11b could be involved in dopaminergic neurodegeneration in the MPTP mouse model of PD. We thus investigated the involvement of DAP12 and CD11b in this pathological process.

**Figure 2:**
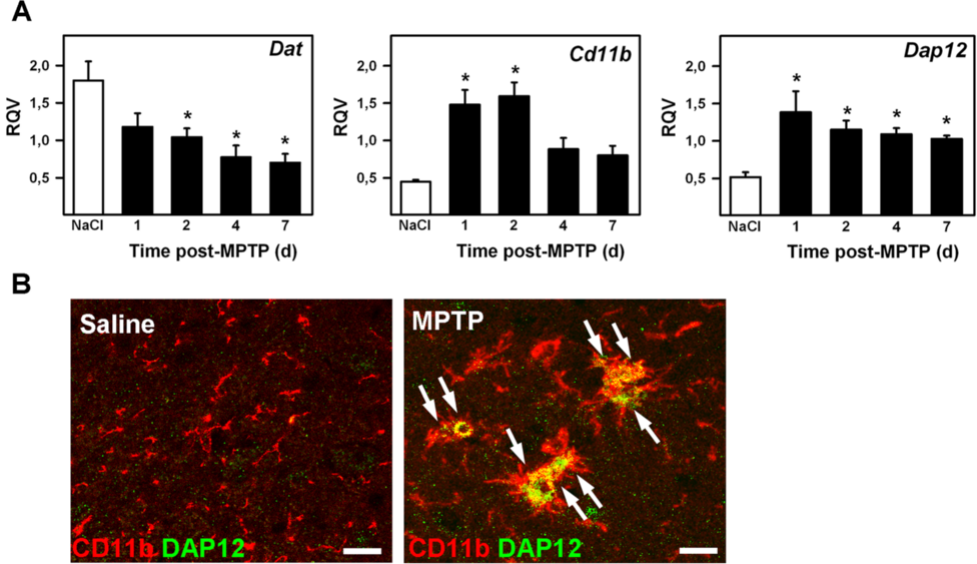
**Microglial expression of Dap12 is induced in the mouse ventral mesencephalon following 1-methyl-4-phenyl-1,2,3,6-tetrahydropyridine (MPTP) intoxication. (A)** Real-time quantitative PCR analysis of *Dat*, *Cd11b* and *Dap12* transcripts in the ventral mesencephalon before (NaCl) and after MPTP intoxication at the indicated times. ^*^P <0.05 compared to NaCl-treated controls; Holm-Sidak post hoc analysis. RQV, relative quantity value; d, days. **(B)** Double immunofluorescent staining for CD11b (red) and DAP12 (green) on mesencephalic tissue sections from control or MPTP-injected mice 2 days after toxin exposure. The arrows indicate hypertrophied and double-labeled microglial cells at the level of the SN *pars compacta*. Scale bar: 30 μm.

We first analyzed by qPCR the time course of *Dap12* and *Cd11b* mRNA expression in the mesesencepalon of mice acutely intoxicated with MPTP. In agreement with the time course of DA neuron injury previously described, we found that dopamine transporter (*Dat*) mRNA level was consistently decreasing from day 1 onward (Figure 2A). In contrast, *Cd11b*mRNA levels significantly increased from the first day after intoxication (Figure 2A). Similarly, analysis of *Dap12* mRNA level revealed a marked three-fold increase one day after MPTP intoxication (Figure 2A). This observation suggests that microglial cell activation following MPTP intoxication may trigger increased DAP12 and CD11b expression in the injured SN. To examine such possibility, we analyzed the cellular expression of DAP12 and CD11b in the SN of control and MPTP-intoxicated mouse by immunostaining. In saline-injected control mice, CD11b-immunoreactive microglial cells were found throughout the SN. These cells displayed a small cell body and ramifications, which are reminiscent of non-activated microglia. In these cells, DAP12 expression was barely detectable (Figure 2B). In MPTP-injected mice, CD11b staining markedly increased in numerous nigral microglial cells of the SN. In some, but not all microglia, a strong DAP12-immunoreactivity could also be detected (Figure 2B). These DAP12-expressing microglia displayed a large cell body and an ameboid-like shape characteristic of a pathological activation state.

### MPTP-induced dopaminergic neuron injury is not attenuated by DAP12 or CD11b deficiency

Our results establish that microglial DAP12 and CD11b contribute to the death of DA neurons *in vitro* upon MPP^+^ exposure. They further show that both DAP12 and CD11b are expressed by microglial cells in the SN when DN are prone to degenerate upon MPTP intoxication. DAP12 and CD11b were also reported to be necessary to induce neuronal death during development. It is therefore tempting to speculate that in the MPTP mouse model of PD, DAP12 and CD11b are involved in the death of DN as well. This hypothesis is further supported by the fact that in a similar model of DN degeneration induced by subchronic injection of MPTP, the death of DN was found to be attenuated in CD11b-deficient mice [5].

**Figure 3:**
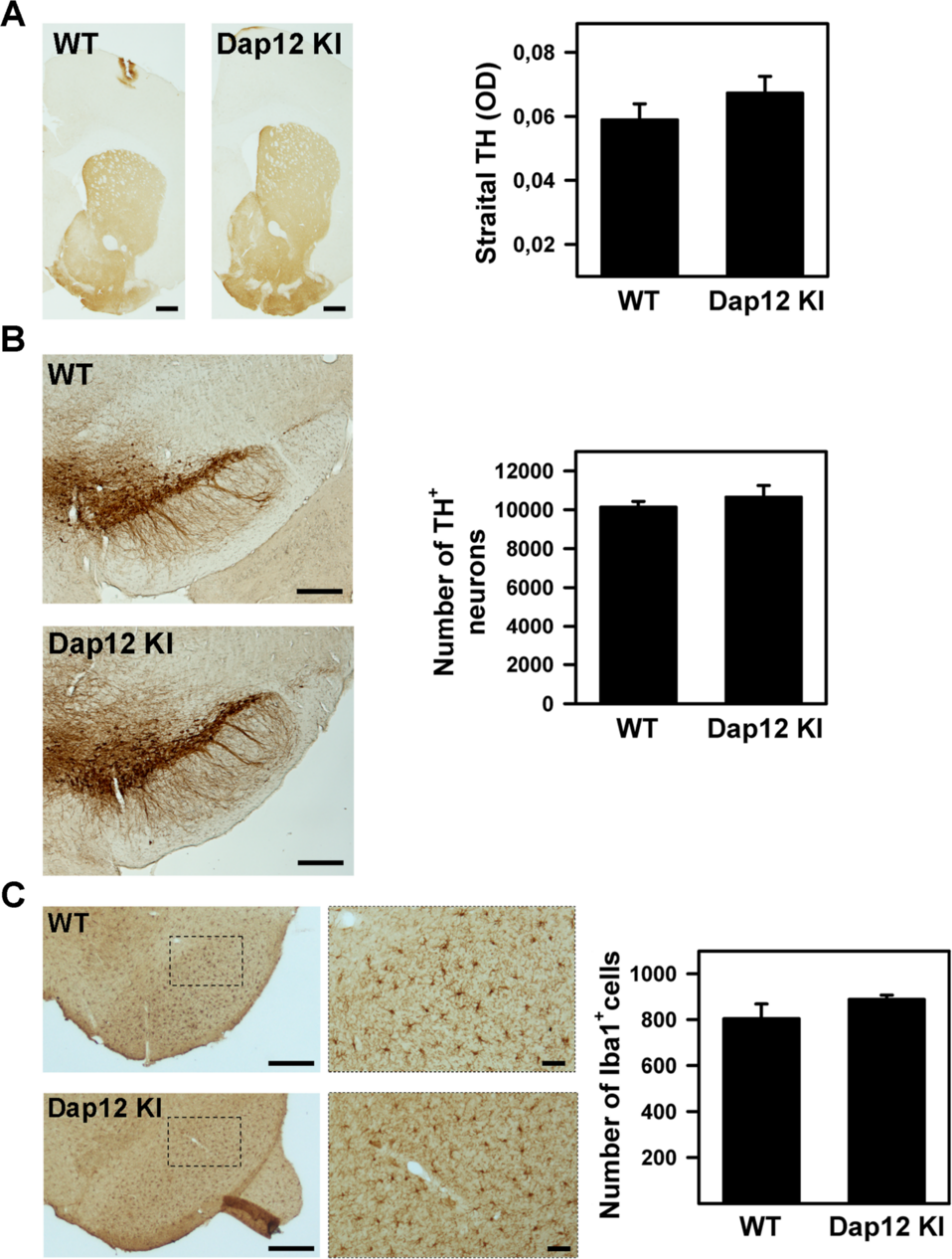
**The nigrostriatal pathway and microglial substantia nigra (SN) content are unaltered in DAP12-deficient mice. (A)** Representative photomicrographs of striatal sections immunostained for tyrosine hydroxylase (TH) from wild-type (WT) and DAP12^KI^ mice. Right panel: quantification of striatal TH immunoreactivity (optical density). Bars represent the mean optical density. Scale bar: 100 μm. **(B)** Representative photomicrographs of mesencephalic sections immunostained for TH from WT and DAP12^KI^ mice. Right panel: quantification of TH^+^ dopaminergic neurons in the SN *pars compacta*. Bars represent the mean number of total nigral TH^+^ neurons. Scale bar: 300 μm. **(C)** Representative photomicrographs of mesencephalic sections from WT and DAP12^KI^ mice and immunostained for the microglial marker Iba1. Dashed boxes delineate the area for higher magnification views shown on the right. Scale bar: left, 400 μm; right, 50 μm. Right panel: quantification of Iba1^+^ microglial cells in the SN *pars compacta* of WT and DAP12^KI^ mice. Bars represent the mean number of Iba1^+^ microglial cells in the SN *pars compacta*.

**Figure 4:**
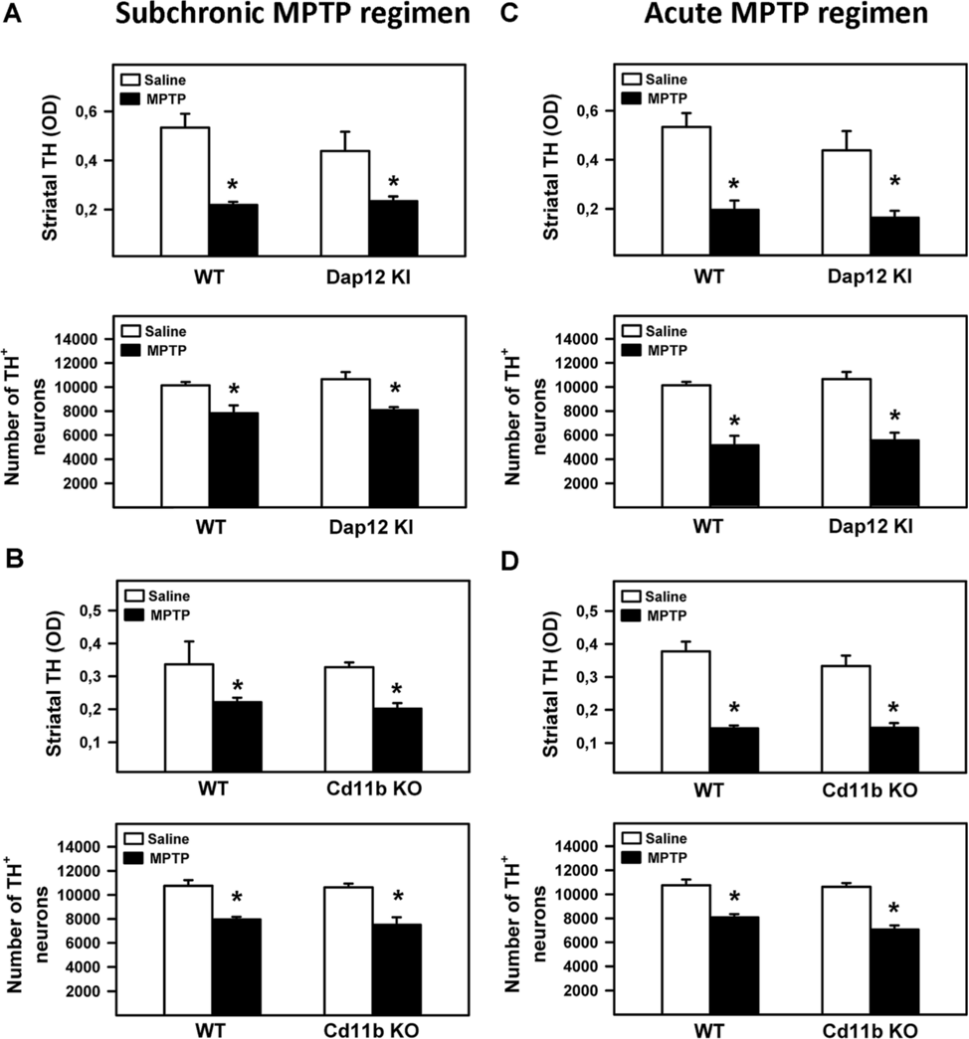
**Mouse deficiency in either DAP12 or CD11b does not mitigate 1-methyl-4-phenyl-1,2,3,6-tetrahydropyridine (MPTP)-induced nigrostriatal pathway injury *in vivo*.** Quantification of striatal tyrosine hydroxylase (TH) immunoreactivity (optical density) and TH^+^ dopaminergic neurons in the SN *pars compacta* from DAP12^KI^ or CD11b KO and their wild-type (WT) littermates at day 21 **(A** and **B**; subchronic regimen of intoxication**)** or 7 **(C** and **D**; acute regimen of intoxication**)** after the last injection of MPTP (black bars) or saline solution (white bars). Irrespective of the mode of intoxication (subchronic or acute), a significant loss of both striatal dopaminergic fibers and nigral DN were observed in all MPTP-treated groups compared to their respective saline controls. No significant differences were found between genotypes. ^*^P <0.001 compared to saline-treated mice, Holm-Sidak post hoc analysis.

Before analyzing the involvement of DAP12 in MPTP-induced neurotoxicity, we checked the integrity of the nigrostriatal pathway in DAP12 mutant mice. We assessed the extent of nigrostriatal pathway injury by quantifying the number of TH-positive neurons in the SN and the level of striatal TH-immunoreactivity as an index of dopaminergic terminal integrity. No significant differences were found in the number of TH-positive neurons and in striatal TH-immunoreactivity between saline-injected DAP12^+/+^ and DAP12^KI^ animals (Figure 3A and B). Likewise, the number and morphology of Iba1^+^-microglial cells in the SN were similar between DAP12^+/+^ and DAP12^KI^ mice (Figure 3C). These results indicate that the development and maintenance of the dopaminergic nigrostriatal pathway is not compromised by DAP12 deficiency. Then, a subchronic regimen of MPTP intoxication was given to DAP12^KI^ and CD11b^−/−^ mice and their WT littermates. Mice were sacrificed 21 days after the last injection. In control DAP12^+/+^ mice, we found that MPTP injection induced a significant death of TH-positive neurons as well as the loss of striatal TH^+^-dopaminergic fibers when compared to saline-injected animals (Figure 4A). Surprisingly however, alteration of TH-positive neurons and TH-positive fibers induced by MPTP injection in the DAP12^KI^ or in the CD11b^−/−^ mutant mice, was the same as those observed in their WT littermates (Figure 4A and B). These results show that DAP12 and CD11b are not involved in the death of DN in the MPTP mouse model of PD using a subchronic mode of intoxication.

These results seem in contradiction with our *in vitro* data and to a previous study showing that CD11b deficiency attenuates the DN death upon sub-chronic intoxication of mice by MPTP. Whether such discrepancy may be related to the mode of dopaminergic injury and intensity of microglial cell activation and neuroinflammation was then tested by applying an acute regimen of MPTP intoxication. In this acute MPTP mouse model, non-cell autonomous mechanisms of neurodegeneration orchestrated by activated microglial cells are known to be involved in the demise of DA neurons [1]. In these conditions, we found that neither DAP12- nor CD11b-deficiency attenuates the loss of DN in the SN or the density of DA fibers in the striatum following MPTP exposure of mice (Figure 4C and D). This was further confirmed by similar decrease in striatal content of dopamine and its metabolites DOPAC and HVA between MPTP-intoxicated DAP12^KI^ and WT mice (Table 1). Importantly, striatal MPP^+^ levels were of the same magnitude between MPTP-intoxicated DAP12^KI^ and WT mice ruling out the possibility that an increase in MPTP metabolism could have masked a putative neuroprotection in DAP12^KI^ animals (Table 2). Taken together, these results show that microglial DAP12 or CD11b deficiencies are able to induce DN neuroprotection *in vitro*, but not *in vivo* in two different MPTP mouse models of PD.

**Table 1:** Striatal monoamine levels (pM/mg tissue)

	**Dopamine**	**DOPAC**	**HVA**
Saline			
DAP12^+/+^(n = 5)	86.1 ± 5.2	13.8 ± 1.7	8.9 ± 1.5
DAP12^KI^ (n = 3)	81.9 ± 10.7	14.6 ± 2.2	9.4 ± 1.1
MPTP			
DAP12^+/+^(n = 5)	5.6 ± 0.9	4.8 ± 0.8	5.2 ± 0.6
DAP12^KI^ (n = 3)	6.5 ± 1.3	4.1 ± 0.5	4.9 ± 0.9

Striatal dopamine, 3,4-dihydroxyphenylacetic acid (DOPAC) and homovanillic acid (HVA) levels in DAP12^+/+^and DAP12^KI^ mice at 7 days after the last MPTP injection do not differ (P >0.05; Mann–Whitney *U*-test) between groups. Data represent means ± SEM for the indicated number of mice. MPTP, 1-methyl-4-phenyl-1,2,3,6-tetrahydropyridine.

**Table 2:** **Striatal 1-methyl-4-phenylpyridinium (MPP**^**+**^**) levels**

	**MPP**^**+ **^**(ng/mg tissue)**
DAP12^+/+^	6.11 ± 2.08
DAP12^KI^	5.86 ± 2.43

Striatal MPP+ levels in DAP12^+/+^ and DAP12^KI^ mice at 90 minutes after a single MPTP injection did not differ (*P* = 0.91; Mann–Whitney *U*-test) between groups. Data represent means ± SEM for five mice per group.

## Discussion

Mounting evidence supports the view that innate immunity orchestrated by microglial cells, the tissue macrophages of the CNS, may contribute to neurodegeneration in PD [1,20]. The microglial response to DN injury sets off deleterious mechanisms among which, induction of catalytic systems that brings about the production of toxic amounts of oxygen-derived and nitrogen-derived species, is thought to play a critical role [1]. This process, known as the oxidative burst, is generally used by phagocytic cells to eliminate invading pathogens. This process has also been described in the CNS when microglial cells trigger the developmental death of supernumerary neurons through the production of superoxide ions [9,11,21,22]. The signaling molecules involved in such a developmental process have shown remarkable similarities with those implicated in the elimination of pathogenic cells by peripheral innate immune cells. Thus, developmental neuronal death in the hippocampus requires the microglial CD11b integrin and DAP12 adaptor protein, which cooperate to promote the production of toxic amounts of superoxide ions (9). Interestingly, prior studies using both *in vitro* and *in vivo* experimental models recapitulating microglial-associated non-cell autonomous mechanisms of dopaminergic cell death, demonstrated that microglial CD11b is likely to play a central role in the demise of DA neurons as well [5]. These data raise the tantalizing hypothesis that developmental mechanisms of microglial-induced neuronal cell death could be reactivated during adulthood in a PD-associated pathological context. In support of this hypothesis, we found that DAP12 expression is strongly stimulated in microglial cells following MPTP-induced nigrostriatal pathway injury whereas it is barely detectable in the SN of saline-injected mice. This lack of DAP12 expression in the normal adult SN is in accordance with previous studies showing that whereas DAP12 could be detected in the hippocampus of E17 mouse embryos, it was not further observed in the brain of 17 day-old mice [11]. Yet, other studies were able to show DAP12 expression mostly in microglial cells and rare neurons in human and mouse cerebral cortex suggesting that under physiological conditions, DAP12 expression may differ from one brain region to another [23]. It is interesting to note that after MPTP intoxication, DAP12 was exclusively detected in hypertrophied and strongly CD11b-labeled microglial cells reminiscent of activated cells. Yet, not all activated CD11b-positive microglial cells expressed DAP12, suggesting that pathological induction of this adaptor signaling molecule is restricted to specific cells with particular functions such as elimination of neurons committed to die [9,11]. In line with a link between DAP12 upregulation and microglial cell activation, the time course of both processes was similar raising the possibility that as previously shown for CD11b [5], DAP12 may represent another important effector function of activated microglial cells during DA neurodegeneration.

We have now shown that microglial DAP12- or CD11b-deficiency is associated with increased DN survival in a mouse *in vitro* model of PD recapitulating microglial-associated non-cell autonomous mechanisms of neuronal cell death. These results confirmed those reported by Hu *et al*. (2008), but also provide further evidence that microglial CD11b and DAP12 may act in concert to induce neurotoxicity as in the developing hippocampus [5,9]. Yet, in contrast to previous studies [3,5], deficiency of microglial CD11b or DAP12 did not change the level of superoxide ions produced by activated microglial cells *in vitro*. Such a difference might be explained by the fact that we directly measured the intracellular microglial superoxide ions whereas in the other studies total extracellular ROS was measured [3,5].

DAP12- and CD11b-deficiency lead to partial neuroprotection of MPP^+^-exposed mesencephalic neurons cultured with microglia. In addition, DAP12 and CD11b were upregulated in nigral microglial cells following MPTP-induced nigrostriatal pathway injury. This prompted us to test the possibility that a microglial CD11b/DAP12 pathway could be involved in non-cell autonomous mechanisms of DN cell death in the MPTP mouse model of PD. Surprisingly, neither DAP12- nor CD11b-deficiency in the mouse were associated with neuroprotection in this experimental model of PD. However, studying the same CD11b^−/−^ transgenic line with the same genetic background, gender and age, it has been found that CD11b-deficient mice were strongly protected against MPTP toxicity [5]. Such divergent results might not be due to the use of different doses of MPTP. Indeed, we used both acute and subchronic regimens of MPTP exposure, corresponding to a cumulative dose of 80 mg/kg and 150 mg/kg of MPTP-HCl, respectively, whereas Hu and collaborators used a cumulative dose of 90 mg/kg. Alternatively, the route of MPTP administration (i.p. versus subcutaneous) could underline different outcomes and will need further investigation. In spite of the uncertainty about the critical role of CD11b in microglial-associated DA cell death *in vivo*, an original finding of our study is that the DAP12 is not crucially involved in the demise of DN in the MPTP mouse model of PD. Therefore, while the developmental CD11b/DAP12 pathway is likely to be reactivated in a PD-like neuropathological context, it is unlikely to play a primary role in neurodegeneration *in vivo*.

It is currently unknown why microglial CD11b or DAP12 deficiency is protective *in vitro* but not *in vivo*. Yet, one may expect that cellular interactions and signaling are much more complex in an integrated biological system than in culture. Hence, inhibition of the CD11b/DAP12 pathway *in vivo* could be compensated by alternative mechanisms, such as activation of Toll-like receptor-4 (TLR4), which is exclusively expressed by microglial cells in the CNS [24]. Previous findings have underlined the role for TLR4 in ‘sterile inflammation’ induced by neuronal damage [25]. Interestingly, among the TLR4 ligands potentially released by dying/suffering neurons during CNS injury, the heat shock protein 60 (Hsp60) which has been implicated in microglia-induced neurotoxicity, is believed to function as an endogenous danger signal to the immune system indicating tissue injury [26]. Since Hsp60 has also been described as an agonist of the DAP12-associated receptor TREM2 [27], one may not exclude the possibility that defective Hsp60/TREM2/DAP12 signaling achieved in DAP12-deficient mice may be substituted by activation of the Hsp60/TLR4 pathway to mediate microglial activation and neurotoxicity *in vivo*. Since activation of the TREM2/DAP12 pathway has been shown to inhibit TLR responses in macrophages [28,29], DAP12 deficiency in MPTP-intoxicated mice could amplify the pathological value of the Hsp60/TLR4 signaling even more, thus masking the putative benefit of DAP12 ablation. Although our *in vitro* data do not support this latter scenario, this will need to be tested *in vivo* in WT and DAP12-deficient mice challenged by LPS infusion into the SN.

## Conclusions

In summary, we have shown that microglial DAP12 and CD11b contribute partially to microglia-associated DN cell death *in vitro* and that DAP12 is reactivated during PD-like nigrostriatal pathway injury induced by MPTP intoxication in adult mice. Yet, genetic ablation of either DAP12 or CD11b in mice does not improve MPTP-associated neuropathological alterations suggesting that this developmental microglia-induced neuronal death mechanism is unlikely to play a significant role in the pathophysiology of PD and should not, therefore, be considered further as a promising therapeutic target for this neurodegenerative disorder.

## Abbreviations

ANOVA: Analysis of variance; CNS: Central nervous system; DAB: 3,3-diaminobenzidine; DHR: Dihydrorhodamine-123; DIV: Days *in vitro*; DMEM: Dulbecco’s modified Eagle’s serum; DN: Dopaminergic neuron; DOPAC: 3,4-dihydroxyphenylacetic acid; EDTA: Ethylenediaminetetraacetic acid; HPLC: High performance liquid chromatography; HVA: Homovanillic acid; ITAM: Immunoreceptor tyrosine-based activation motif; LPS: Lipopolysaccharide; MPP+: 1-methyl-4-phenylpyridinium; MPTP: 1-methyl-4-phenyl-1,2,3,6-tetrahydropyridine; NAPDH: Nicotinamide adenine dinucleotide phosphate-oxidase; PBS: Phosphate-buffered saline; PD: Parkinson’s disease; PFA: Paraformaldehyde; qPCR: Quantitative polymerase chain reaction; ROS: Reactive oxygen species; RNS: Reactive nitrogen species; SEM: Standard error of the mean; SN: Substantia nigra; TH: Tyrosine hydroxylase; TLR4: Toll-like receptor-4; WT: Wild-type.

## Competing interests

The authors declare that they have no competing interests.

## Authors’ contributions

KK carried out the *in vivo* studies including mouse perfusion, brain processing, tissue immunohistochemistry, cell counting, double immunofluorescent staining on mouse brain tissue sections and data analysis, helped to set up *in vitro* models, performed the statistical analysis and draft and wrote the manuscript. YM set up the *in vitro* models, carried out cell cultures and treatments, cell immunostaining and counting, and performed data analysis. CB performed immunofluorescent staining on mouse brain tissue sections, image acquisition, and wrote the manuscript. DAF performed HPLC analysis. ECH gave a critical reading of the manuscript. AB participated in data analysis, draft and wrote the manuscript. SH conceived the study, participated in its design and coordination, performed MPTP intoxications, drafted and wrote the manuscript. All authors read and approved the final manuscript.

## Authors’ information

KK present address: UPMC University Paris 06, CNRS, UMR7102, Team Development and Aging of the Nervous System, F-75005 Paris, France. ECH, AB and SH are investigators at the Centre National de la Recherche Scientifique (CNRS).
